# AI-based lumbar central canal stenosis classification on sagittal MR images is comparable to experienced radiologists using axial images

**DOI:** 10.1007/s00330-024-11080-0

**Published:** 2024-09-20

**Authors:** Jasper W. van der Graaf, Liron Brundel, Miranda L. van Hooff, Marinus de Kleuver, Nikolas Lessmann, Bas J. Maresch, Myrthe M. Vestering, Jacco Spermon, Bram van Ginneken, Matthieu J. C. M. Rutten

**Affiliations:** 1 Diagnostic Image Analysis Group, Radboud University Medical Center, Nijmegen, The Netherlands; 2 Department of Orthopedics, Radboud University Medical Center, Nijmegen, The Netherlands; 3 Department of Research, Sint Maartenskliniek, Nijmegen, The Netherlands; 4 Department of Radiology, Hospital Gelderse Vallei, Ede, The Netherlands; 5 Department of Radiology, Jeroen Bosch Hospital, ‘s-Hertogenbosch, The Netherlands

**Keywords:** Spine, MRI, Lumbar central canal stenosis, Machine learning, Deep learning

## Abstract

**Objectives:**

The assessment of lumbar central canal stenosis (LCCS) is crucial for diagnosing and planning treatment for patients with low back pain and neurogenic pain. However, manual assessment methods are time-consuming, variable, and require axial MRIs. The aim of this study is to develop and validate an AI-based model that automatically classifies LCCS using sagittal T2-weighted MRIs.

**Methods:**

A pre-existing 3D AI algorithm was utilized to segment the spinal canal and intervertebral discs (IVDs), enabling quantitative measurements at each IVD level. Four musculoskeletal radiologists graded 683 IVD levels from 186 LCCS patients using the 4-class Lee grading system. A second consensus reading was conducted by readers 1 and 2, which, along with automatic measurements, formed the training dataset for a multiclass (grade 0–3) and binary (grade 0–1 vs. 2–3) random forest classifier with tenfold cross-validation.

**Results:**

The multiclass model achieved a Cohen’s weighted kappa of 0.86 (95% CI: 0.82–0.90), comparable to readers 3 and 4 with 0.85 (95% CI: 0.80–0.89) and 0.73 (95% CI: 0.68–0.79) respectively. The binary model demonstrated an AUC of 0.98 (95% CI: 0.97–0.99), sensitivity of 93% (95% CI: 91–96%), and specificity of 91% (95% CI: 87–95%). In comparison, readers 3 and 4 achieved a specificity of 98 and 99% and sensitivity of 74 and 54%, respectively.

**Conclusion:**

Both the multiclass and binary models, while only using sagittal MR images, perform on par with experienced radiologists who also had access to axial sequences. This underscores the potential of this novel algorithm in enhancing diagnostic accuracy and efficiency in medical imaging.

**Key Points:**

***Question*** *How can the classification of lumbar central canal stenosis (LCCS) be made more efficient?*

***Findings*** *Multiclass and binary AI models, using only sagittal MR images, performed on par with experienced radiologists who also had access to axial sequences.*

***Clinical relevance*** *Our AI algorithm accurately classifies LCCS from sagittal MRI, matching experienced radiologists. This study offers a promising tool for automated LCCS assessment from sagittal T2 MRI, potentially reducing the reliance on additional axial imaging*.

## Introduction

Lumbar central canal stenosis (LCCS) is a condition where the spinal canal is narrowed, resulting in compression of the dural sac, possibly causing low back pain and radiating pain [1–3]. It has a prevalence of 11% in the general population and is most often observed in elderly patients [4, 5]. Magnetic resonance imaging (MRI) is primarily used to diagnose LCCS since it provides excellent soft tissue contrast, enabling a clear distinction of the intervertebral discs (IVDs), nerves, and the osseous spinal canal [6]. Grading systems such as the Lee’s and the Schizas grading systems have been developed to consistently and reliably classify the severity of LCCS [7–10]. Alternatively, several studies have proposed quantitative diagnostic criteria with cutoff values for different severities of LCCS [11]. Utilizing quantitative measurements provides a more objective approach to classifying LCCS, minimizing ambiguity. However, manual quantitative measurements are time-consuming and are, therefore, rarely used in routine clinical practice [7].

Despite its efficacy in diagnosing LCCS, MRI has its limitations. Notably, MRI is expensive and time-consuming, often requiring multiple sequences to obtain axial images at different vertebral levels, orthogonal to the spinal canal. This can lead to situations where certain vertebral levels lack axial images or where the images obtained are not precisely parallel to the IVD. Consequently, developing an automatic grading system that relies solely on sagittal images for evaluating LCCS across all levels would be beneficial. Such a system would streamline the diagnostic process by automatically measuring LCCS, which reduces reporting time while ensuring objective and consistent evaluation of the condition’s severity.

Several deep learning systems have been developed for grading LCCS automatically, using methods such as convolutional neural networks [12–16]. However, employing neural networks for LCCS classification poses challenges in terms of interpretability and explainability of predictions. Integrating additional automatic quantitative measurements could provide further insight into the classification process. A recent study by Bharadwaj et al addressed this issue by developing a decision tree classifier to predict LCCS classifications, using the ratio between the cross-sectional areas of the dural sac and the IVDs [17]. However, this classifier solely relied on one novel metric, overlooking other conventional metrics with established correlations to LCCS, such as the dural sac AP-diameter and cerebrospinal fluid (CSF) signal loss [8, 18]. Incorporating these metrics could enhance the classifier’s robustness and comprehensiveness, offering a better understanding of LCCS severity and supporting clinical decision-making.

The purpose of this study was to develop and validate a novel algorithm that classifies LCCS. This algorithm automatically extracts quantitative metrics related to LCCS based on 3D segmentations, which are used to classify LCCS on lumbar sagittal MRI scans.

## Methods

### Data selection

Lumbar spine MRI studies were collected at the Radboud University Medical Center in the Netherlands. The complete dataset consisted of all MRI studies of patients suffering from low back pain and/or neurogenic leg pain based on the MRI study description, acquired between 2014 and 2019 on a 1.5-T Siemens MRI system. This retrospective study was approved by the institutional review board (IRB 2016-2275). Exemption from informed consent was granted due to the use of retrospectively deidentified MRI examinations.

The following exclusion criteria were applied:

- No LCCS based on the radiology report. Exclusion was performed by including MRI studies with reports that include “canal stenosis”, and by excluding MRI studies with reports containing “no canal stenosis” or “no spinal canal stenosis”.
- Studies that do not contain at least one T2 sagittal and one T2 axial series.
- Studies where accurate LCCS assessment was deemed infeasible due to, for example, post-operative changes, metastases, or poor image quality.

**Fig. 1:**
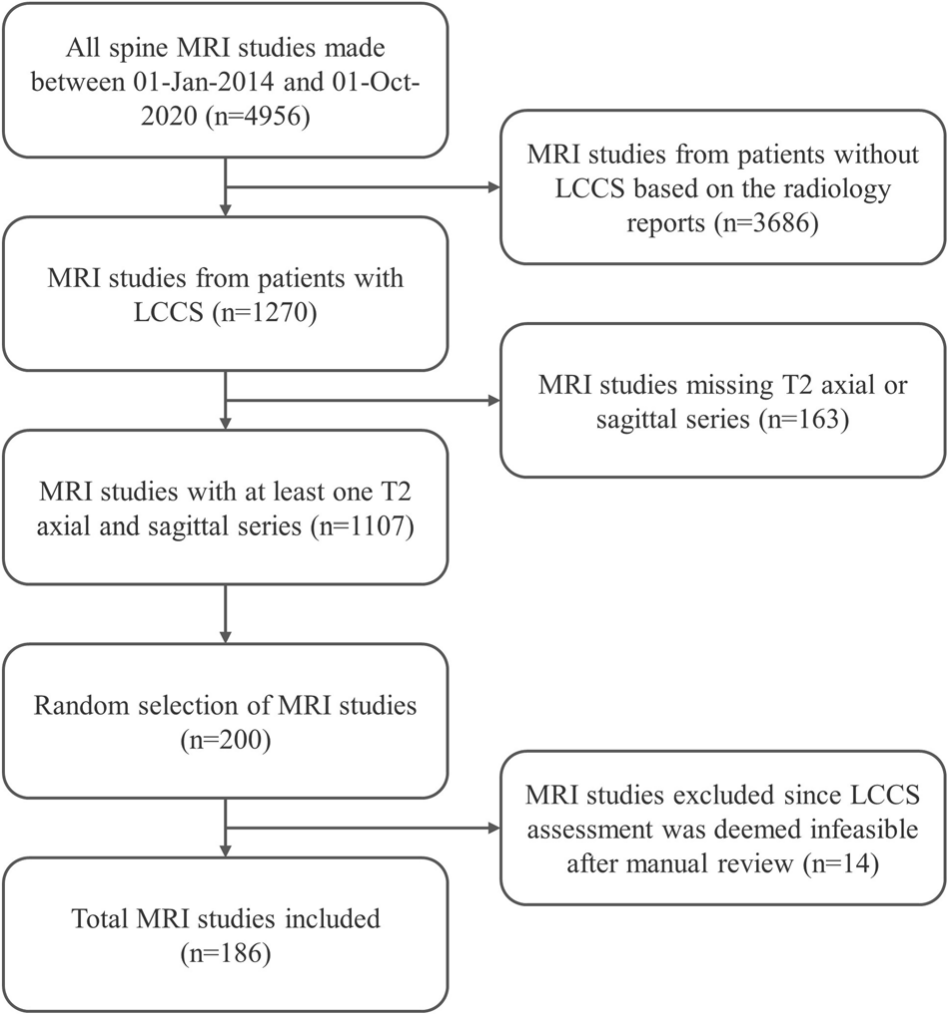
Flow diagram of MRI study selection

This resulted in 1107 eligible MRI studies, of which a random subset was drawn. Given the labor-intensive nature of data annotation, a sample size of 200 studies was selected. If a patient received more than one MRI examination, only the oldest MRI study was included to avoid post-treatment scans. The exclusion of post-operative scans was motivated by the often-compromised visibility caused by the presence of scar tissue and metal artifacts, which can significantly hinder accurate LCCS assessment. All 200 MRI studies underwent manual review by experienced radiologists (reader 1 and 2 (R1 and R2)) to assess the feasibility of accurately evaluating LCCS. Subsequently, 14 MRI studies were excluded from the analysis: eleven were excluded due to post-operative changes, one due to the presence of metastases, one due to misalignment between the sagittal and axial sequences, and one was misclassified as a lumbar acquisition. In total, 186 MRI studies from unique patients suspected of LCCS were included, resulting in a total of 312 axial T2 series of various vertebral levels, and 186 sagittal T2 series (Fig. 1).

In accordance with the CLAIM 2024 (Checklist for Artificial Intelligence in Medical Imaging) guidelines [19], we ensured comprehensive reporting and transparency throughout the study. Detailed adherence to each item in the CLAIM 2024 checklist is documented in Appendix A.

### Manual data labeling

All MRI studies underwent independent manual labeling by four experienced radiologists: R1, R2, R3, and R4, with respectively 27, 25, 15, and 14 years of experience. The severity of LCCS was assessed on both axial and sagittal images using the classification system described by Lee et al with grade 0 being ‘no stenosis,’ grade 1 ‘mild stenosis,’ grade 2 ‘moderate stenosis,’ and grade 3 ‘severe stenosis’ [8]. The highest observed grade was annotated for each IVD level visible in the available T2 axial images. Additionally, a mid-sagittal image containing disk numbers was included to ensure consistent identification of discs.

Following individual readings, R1 and R2 conducted a second consensus reading for all the IVD levels where they had assigned different grades. This consensus reading was regarded as the ground truth. The grading process was executed on the medical imaging annotating platform Grand-Challenge.org.

For binary classification, the grades from the consensus reading were ‘binarized’. Lee grades 0 and 1 were considered indicative of ‘no stenosis’ and grades 2 and 3 were classified as ‘stenosis’.

### Deep learning segmentation

**Fig. 2:**
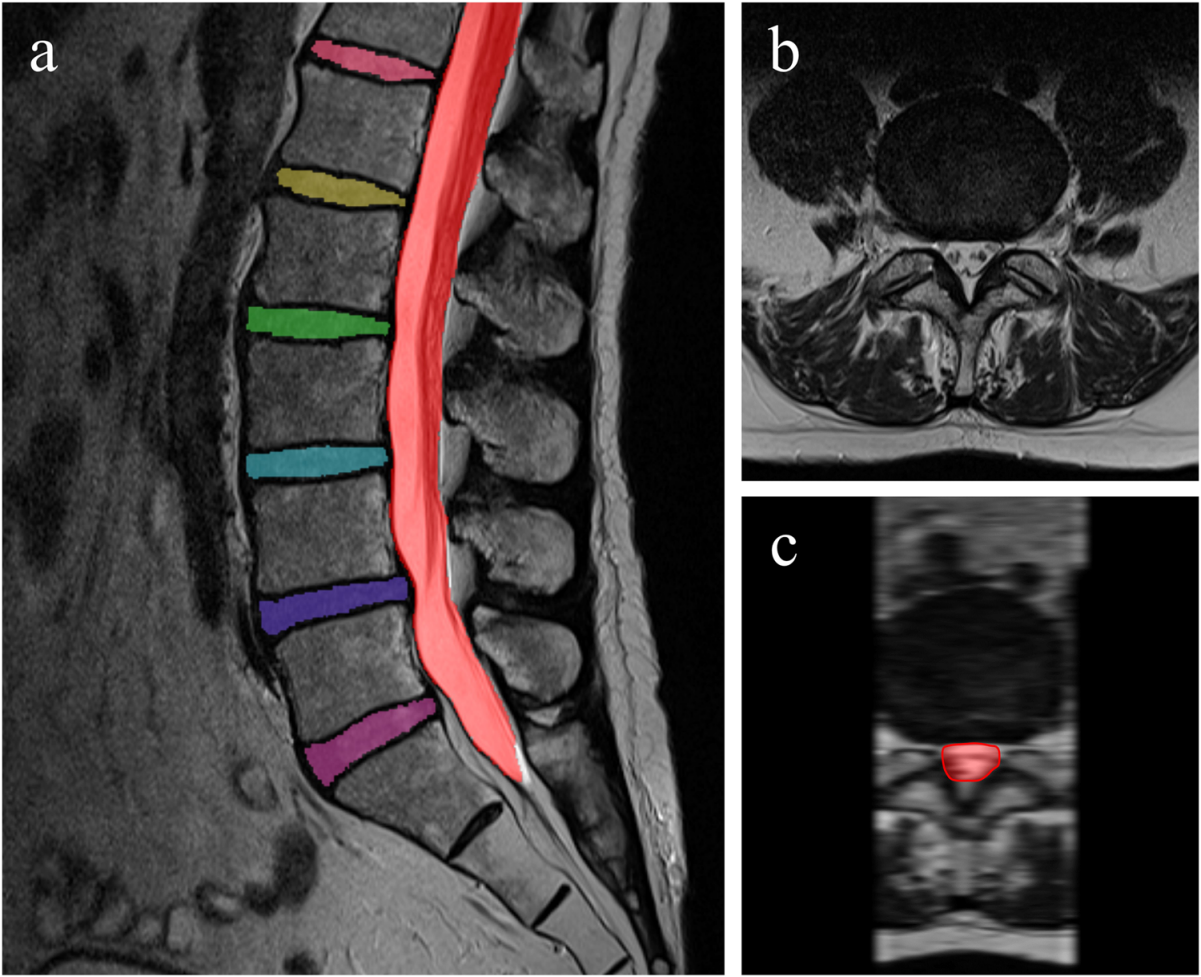
An MRI study paired with its automatically generated segmentation mask is presented. **a** Depicts the mid-sagittal slice from a series of sagittal T2-weighted MRI images. **b** Illustrates an axial slice at the L4-L5 intervertebral disc level. **c** Displays a reconstructed axial view, derived from the sagittal MRI series, corresponding to the level of image **b**

An existing lumbar spine segmentation algorithm, capable of segmenting the vertebrae, IVDs, and spinal canal, was employed to generate the required segmentation masks [20]. This U-net-based algorithm uses a 3D patch-based iterative scheme to segment one pair of vertebrae and the corresponding inferior IVD at a time, together with the segment of the spinal canal covered by the image patch [20]. It is trained and validated on the publicly available multicenter SPIDER dataset, which includes 447 sagittal lumbar MRI series from 218 patients with low back pain [21]. The reported Dice score of the spinal canal and IVD segmentation were 0.92 (SD 0.04) and 0.84 (SD 0.10) with 100% and 98.7% detection, respectively, in sagittal T2 scans. The segmentation of the spinal canal corresponds to the anatomical dural sac. An example of an automatically generated segmentation mask is illustrated in Fig. 2. Additional examples of successful as well as faulty segmentation masks can be found in Appendix B.

### Automatic measurements

The following metrics were automatically extracted using the segmentation masks:

1. Dural sac cross-sectional area (CSA), defined as the cross-sectional area of the dural sac measured in an axial view of the spine (Fig. 3). The CSA, expressed in square millimeters, was calculated by summing up the surface area of all voxels of the spinal canal mask.

**Fig. 3:**
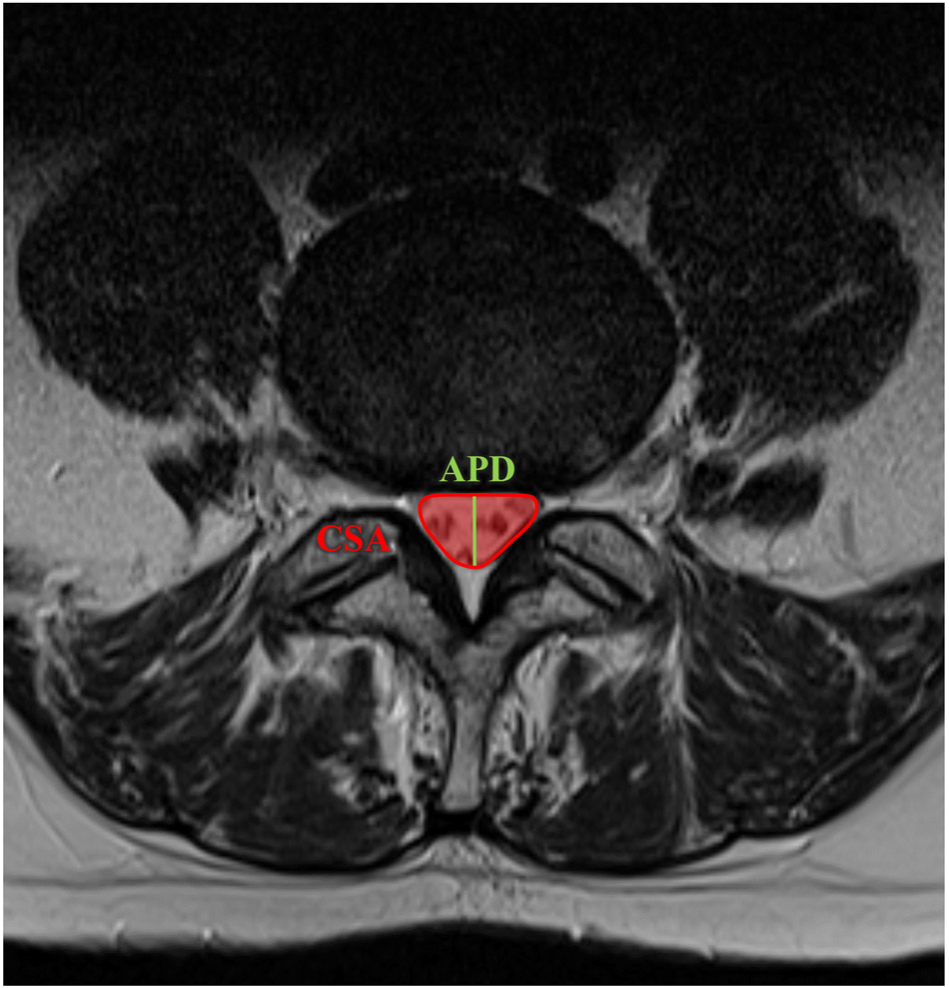
Visualization of automatically generated measurements, which includes the dural sac antero-posterior diameter (APD) in green and the dural sac cross-sectional area (CSA) in red
2. Dural sac antero-posterior diameter (APD), expressed in millimeters, was defined as the largest possible antero-posterior distance of the spinal canal mask (Fig. 3).
3. CSF signal loss (FSL) was calculated by determining the average signal strength of all MRI voxels within the spinal canal mask of an angled axial plane.

**Fig. 4:**
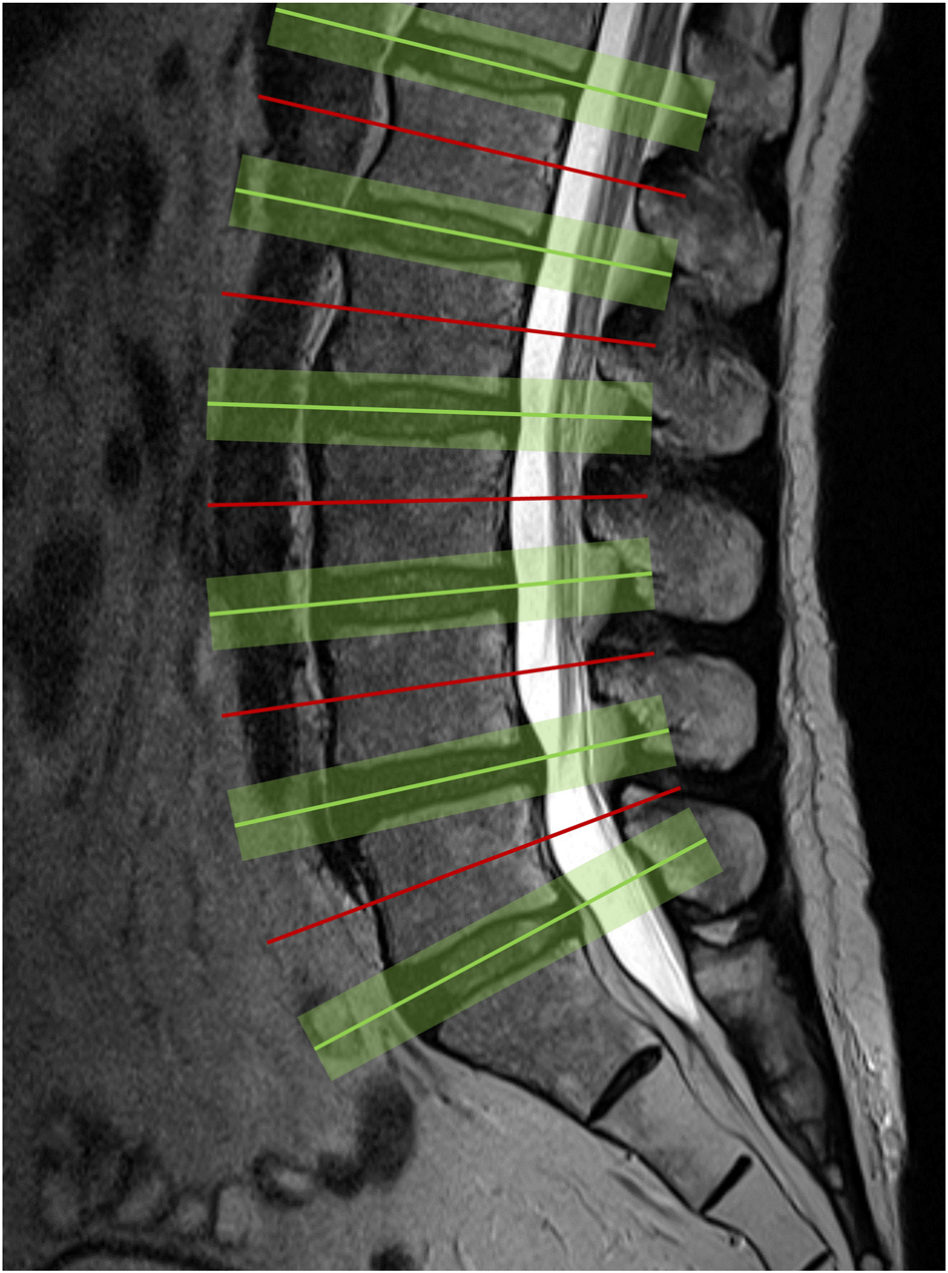
Visualization of the angulations of all measurements. Angulation was determined for each intervertebral disc, with absolute measurements extracted from the green volumes, utilizing only the measurements from the most stenotic plane within that volume. These absolute measurements were then compared to the cranially adjacent mid-vertebral measurements (presented by red lines) to derive the relative measurement values (the ratio-based values)

To accurately perform these measurements, the axial view of the spinal canal should be parallel to the IVD of that specific level. This is accomplished by rotating and resampling the MRI volume separately for each IVD level (Fig. 4). The 3D spatial angulation required for rotating and resampling the MRI volume was determined by fitting a 3D plane through each IVD using principal component analysis (PCA) [22]. The third axis of the PCA, which is perpendicular to the 3D plane, was used to determine the angulation of the IVD. The middle slice of each IVD, alongside 20 slices above and below the middle slice, was extracted for automatic measurements. With a standardized slice-thickness of 0.5 mm this was equal to a total range of 2 cm around the center of each IVD. This range was chosen to ensure that an average healthy IVD is entirely included in the measurements and that with degenerative pathological IVDs the endplates are incorporated as well. Measurements of the most caudal 15 mm of the spinal canal segmentation were discarded. This was based on the naturally decreased size of the dural sac in this area, which could lead to false positives for stenoses if included.

The three metrics (CSA, APD, and FSL) were quantified across all 40 axial planes for each IVD, orthogonal to the spinal canal. Subsequently, only the measurements of the most stenotic plane were utilized. The most stenotic plane was determined through a cumulative ranked system that integrates all three metrics. Axial planes were individually ranked for each metric, and the plane with the lowest cumulative rank was identified as the most stenotic. Only the measurements from this most stenotic plane were used for LCCS classification.

For each of the three aforementioned metrics, two measurements were taken, an absolute value and a ratio-based value. These additional relative metrics were included to correct for size differences between vertebral levels, as well as anatomical differences between patients. The ratios were calculated between the IVD level measurements and the corresponding reference measurements (Fig. 4). Reference measurements were taken at the mid-vertebral level of each cranially adjacent vertebra for all IVDs. The angulation of the reference measurement was computed by taking the average of the angulations of the two neighboring IVDs. Examples of the angled IVD planes and the corresponding reference measurements are shown in Fig. 4. These relative metrics are reported as rCSA, rADP, and rFSL.

### Training data

The total dataset consists of 683 IVD levels from 186 patients suspected of LCCS. For each IVD level, the dataset includes six automatic measurements alongside the ground truth Lee grade, serving as the label. Table 1 provides a comprehensive overview of the entire dataset.

**Table 1:** Patient demographics and clinical characteristics

MRI studies included	186
Age in years, mean (SD)	61.4 (13.8)
Gender male, *n* (%)	105 (56.5)
Patients with stenosis present (Lee grade > 0), *n* (%)	148 (79.6)
Number of IVD levels per Lee grade in consensus reading, *n* (%)	683 (100)
Grade 0	317 (46.4)
Grade 1	169 (24.7)
Grade 2	114 (16.7)
Grade 3	83 (12.2)
IVD levels^a^ per anatomical level, *n* (%)	683 (100)
T12-L1	12 (1.7)
L1-L2	50 (7.3)
L2-L3	105 (15.4)
L3-L4	173 (25.3)
L4-L5	178 (26.1)
L5-S1	165 (24.2)

*SD* standard deviation, *IVD* intervertebral disc

^a^ Anatomical levels were determined by AI segmentation algorithm, which was kept consistent between manual and automatic measurements

### Classification

During the development phase, experiments were conducted with several machine learning models, including logistic regression, decision trees, and random forests, ultimately selecting random forests based on superior performance. The results of these experiments are shown in Appendix C. Two random forest classification models, one for multiclass and one for binary classification, were developed using scikit-learn 1.3.2 [23]. Both models underwent training via tenfold cross-validation, employing a 90-10 training-validation split strategy. The binary model utilized a standard tenfold split, whereas the multiclass model employed a stratified tenfold split to address discrepancies in class sizes.

The random forest models were trained under specific settings chosen after performing a random grid search. For the multiclass classification, the parameters were set to 1 sample per leaf, 208 trees, and a maximum depth of 46, whereas for binary classification, they were set to 23 samples per leaf, 29 trees, and a maximum depth of 72.

The multiclass model was trained with the consensus reading grades as labels and the six specified metrics as features. Conversely, the binary model utilized binarized consensus reading grades alongside the same six metrics. To assess the impact of individual metrics on model performance an ablation study was done. Evaluations were conducted with varying feature subsets, deliberately omitting certain metrics in each iteration.

### Statistical analysis

The performance of the multiclass random forest model, in comparison to the ground truth, was quantified using Cohen’s quadratic weighted kappa score (κw). Levels of agreement were interpreted as: κw ≤ 0 ‘poor’ agreement, 0.01–0.20 ‘slight,’ 0.21–0.40 ‘fair,’ 0.41–0.60 ‘moderate,’ 0.61–0.80 ‘substantial,’ and 0.81–1.00 ‘almost perfect’ agreement [24, 25]. The κw was calculated based on the predictions on the validation sets during tenfold cross-validation. The κw of R3 and R4, compared with the consensus reading, were calculated and used to compare the model’s performance with the radiologists’ performance.

The performance of all binary random forest models was evaluated using the area under the receiver operating characteristic curve (AUC). AUC values were interpreted as: 0.50–0.59 ‘fail,’ 0.60–0.69 ‘poor,’ 0.70–0.79 ‘fair,’ 0.80–0.89 ‘good,’ and 0.90–1 ‘excellent’ [26]. To compute this metric, probability predictions generated from the validation folds during cross-validation were compared to the ground truth. Similarly, the binarized R3 and R4 gradings were compared to the ground truth to calculate the sensitivity and specificity. These metrics served as a benchmark to compare the binary model’s performance against that of radiologists. Not only were the optimal sensitivity and specificity of the model (determined by the Youden’s J index [27]) used for the comparison, but also the sensitivities and specificities thresholded at matching performance of the two readers. Lastly, the accuracy, positive predictive value (PPV), and negative predictive value (NPV) were computed for the algorithm (thresholded at the Youden’s J index) and the two readers.

## Results

**Fig. 5:**
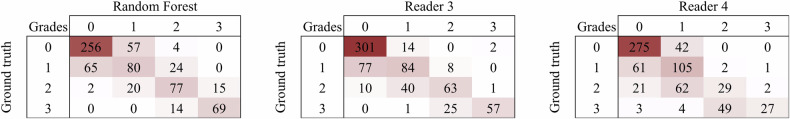
Confusion matrices of the multiclass random forest model, Reader 3, and Reader 4. The results are compared to the ground truth, which is the consensus reading

Cross-validation of the multiclass random forest model resulted in a mean κw of 0.86 (95% CI: 0.82–0.90). R3 and R4 obtained κw scores of 0.85 (95% CI: 0.80–0.89) and 0.73 (95% CI: 0.68–0.79), respectively. The confusion matrices of the algorithm, R3, and R4 are shown in Fig. 5.

**Fig. 6:**
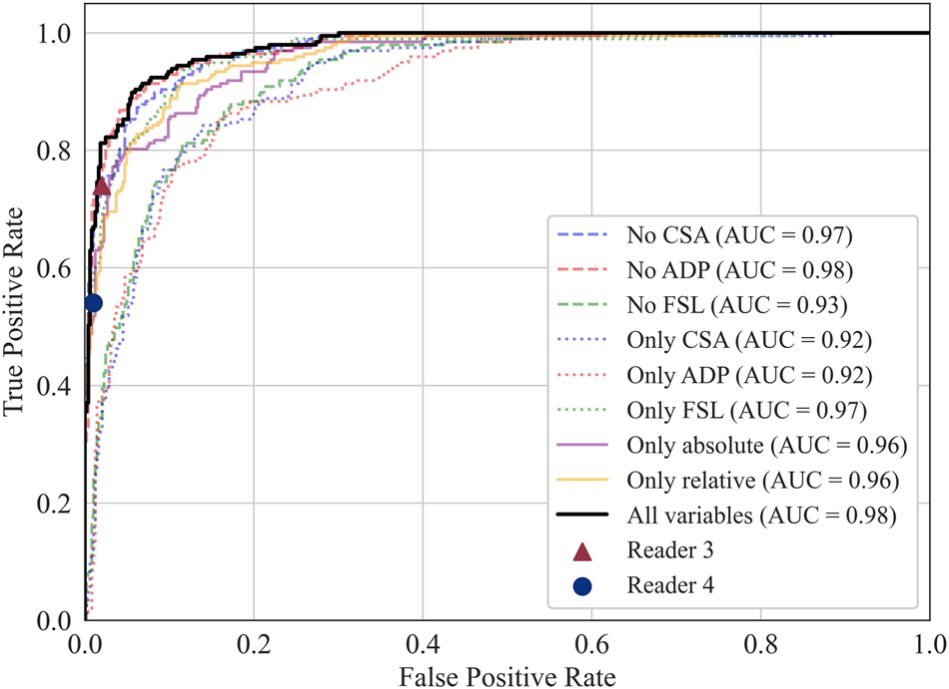
Receiver operating characteristic (ROC) curves of all binary models with different input metrics. The performances of Reader 3 and 4 are also shown, compared to the consensus. CSA, cross-sectional area; APD, antero-posterior diameter; FSL, fluid signal loss; AUC, area under the curve

The mean AUC during cross-validation of the binary random forest model was 0.98 (95% CI: 0.97–0.99) compared to binarized consensus grades. Analysis of the model’s performance at the optimal threshold of 0.46, determined by maximizing Youden’s J index, resulted in a sensitivity of 93% (95% CI: 91–96%) and a specificity of 91% (95% CI: 87–95%) with a J index of 0.84. R3 and R4 obtained specificity scores of 98 and 99% and sensitivity scores of 74 and 54%, respectively. Table 2 shows the sensitivity and specificity of the binary model thresholded at the closest matching sensitivity and specificity of the two readers. Figure 6 shows the receiver operating characteristic (ROC) curve of all binary models, including the performance of R3 and R4. Finally, the algorithm demonstrated an accuracy of 0.93, a PPV of 0.84, and an NPV of 0.96. In comparison, R3 and R4 showed accuracies of 0.91 and 0.86, PPVs of 0.94 and 0.97, and NPVs of 0.90 and 0.84, respectively.

**Table 2:** Sensitivities and specificities of the binary model matched to the readers

	Sensitivity	Specificity
Reader 3	0.741	0.979
Binary model at closest matching sensitivity	0.746	0.986
Binary model at closest matching specificity	0.772	0.982
Reader 4	0.543	0.994
Binary model at closest matching sensitivity	0.503	0.996
Binary model at closest matching specificity	0.629	0.994
Binary model at Youden’s index	0.933	0.910

To test the contribution of the various metrics included in the presented models, an ablation study was performed. Table 3 shows performance of the various multiclass and binary models when certain features are removed from the training data.

**Table 3:** Feature contribution

Variables included	Multiclass performance (κw (95% confidence interval))	Binary performance (AUC (95% confidence interval))
No CSA^a^	0.84 (0.80–0.88)	0.97 (0.96–0.99)
No ADP^a^	0.84 (0.80–0.89)	0.98 (0.97–0.99)
No FSL^a^	0.72 (0.66–0.77)	0.93 (0.91–0.94)
Only CSA^a^	0.68 (0.62–0.74)	0.92 (0.90–0.94)
Only APD^a^	0.69 (0.63–0.75)	0.92 (0.89–0.94)
Only FSL^a^	0.82 (0.78–0.87)	0.97 (0.95–0.98)
Only absolute	0.77 (0.74–0.80)	0.96 (0.95–0.97)
Only relative	0.81 (0.78–0.84)	0.96 (0.95–0.98)
All variables	0.86 (0.82–0.90)	0.98 (0.97–0.99)

*CSA* cross-sectional area, *APD* antero-posterior diameter, *FSL* fluid signal loss, *AUC* area under the curve

^a^ Both the absolute as well as the relative measurements of this variable are removed during training

## Discussion

This study introduces a new AI-based algorithm designed to accurately classify LCCS at any IVD level, employing only sagittal lumbar MR images. The findings demonstrate almost perfect agreement with expert radiologists’ consensus, which was graded using both sagittal and axial MR images. The multiclass model showed Cohen’s weighted kappa (κw) score of 0.86 (95% CI: 0.82–0.90), while the binary model (grade 0 and 1 ‘no stenosis’ and grades 2 and 3 ‘stenosis’) exhibits a sensitivity of 93% (95% CI: 91–96%) and specificity of 91% (95% CI: 87–95%). Comparative analysis with two radiologists reveals compelling results: the multiclass grading yields κw scores of 0.85 (95% CI: 0.80–0.89) and 0.73 (95% CI: 0.68–0.79), while binary grading demonstrates specificity scores of 98 and 99%, and sensitivity scores of 74 and 54%, respectively. Finally, the binary model demonstrated an accuracy of 0.93, compared to 0.91 and 0.86 for R3 and R4, with comparable PPVs and NPVs. These findings underscore a remarkable alignment between both AI models and radiologists’ consensus gradings, affirming the efficacy of the proposed approach.

Previous studies have focused on LCCS detection and grading, utilizing various AI methods. Many of these studies relied on opaque black box models, such as convolutional neural network classifiers, yielding κw scores ranging from 0.80 to 0.82 in multiclass classification and AUC values of 0.95–0.97 in binary classification [12–16]. In contrast, our approach emphasizes explainability and efficiency by employing a more understandable methodology that uses quantitative measurements while requiring less training data. By leveraging these measurements, our model offers traceable predictions, enhancing interpretability [28].

Bharadwaj et al developed a decision tree model based on the ratio between the cross-sectional areas of the dural sac and the IVD, with axial MRI slices as input [17]. Their model achieved a κw of 0.80 (90% CI: 0.76–0.82), lower than our method, which exclusively utilizes sagittal MR images. Moreover, our research dataset exhibits a higher prevalence of moderate to severe LCCS grades, accounting for 28% of cases, in contrast to Bharadwaj’s dataset, which only compromised 15% of such cases. This highlights the robustness and applicability of our approach across a broad spectrum of LCCS severity levels.

A notable strength of our method is that it leverages six distinct measurements related to LCCS, namely the absolute and relative surface area (CSA), AP-diameter (APD), and signal intensity (FSL) of the dural sac [11, 18]. These measurements, while informative, are labor-intensive and, therefore, rarely conducted in clinical settings [7]. Our algorithm automatically performs these measurements at any specified level of the lumbar spine. Among the metrics included, FSL emerged as the most influential factor in automatic prediction. Despite the pivotal role FSL plays in determining the Lee grade [8], and its demonstrated effectiveness in LCCS assessment [18], to our knowledge no research has been published on using automatic FSL measurements for LCCS classification. This underscores the novelty and potential impact of our approach in advancing LCCS diagnosis and management.

Furthermore, by exclusively utilizing sagittal lumbar MRI scans as input, our algorithm can automatically classify LCCS across all vertebral levels without the need for axial sequences. In clinical practice, axial sequences are angulated such that the MR volume is parallel to the IVD. However, in cases where multiple IVD levels are encompassed within the same MR volume, achieving parallel alignment for all IVDs becomes challenging due to the spine’s natural curvature. Our approach addresses this challenge by aligning the MRI volume parallel to the segmented IVD in 3D. This method of extracting IVD angulation was successfully used in a recent study on automatic Cobb angle measurements, demonstrating comparable accuracy to human readers [29].

This study has several limitations. First, the absence of an external test set for validating the model is notable. The reliance on data solely from one hospital for the tenfold cross-validation may restrict the broader applicability of the findings. Furthermore, even though the prevalence of stenotic IVD levels is higher compared to other studies, the size of the dataset is relatively small, possibly limiting the generalizability. Especially when looking at the T12-L1 IVD level or higher. To ensure the robustness of the algorithm, it should be trained on more data, which includes post-operative MRI scans and should be sourced from different MRI scanners across various hospitals. In this study, we demonstrated that this novel method is effective with the current dataset. However, future research should focus on testing the presented algorithm on larger multicenter datasets to ensure its robustness across diverse patient populations and imaging conditions.

Second, while the presented algorithm accurately classifies LCCS, it does not encompass other pathologies often associated with LCCS, such as lateral recess stenosis and foraminal stenosis. Given their significance in treatment decision-making, accurate assessment of these pathologies is essential. A potential solution could involve developing a model where the width of the neural foramina is measured based on vertebrae and IVD segmentation masks, which would require additional research.

Last, relying solely on sagittal MR images inevitably means working with lower axial resolutions compared to axially obtained MR images. This may potentially reduce the precision of axial measurements compared to those derived from axial MR images. However, despite this limitation, our study demonstrates that the lower axial resolution remains adequate for accurate LCCS classification.

In summary, this study introduces a novel AI-based algorithm designed for both multiclass and binary LCCS classification, demonstrating performance comparable to experienced radiologists. Through the integration of automatic quantitative measurements, our approach not only achieves high accuracy but also enhances the interpretability and traceability of LCCS predictions, distinguishing it from opaque models. The algorithm presented holds promise as a valuable tool for automating LCCS assessment from sagittal T2 MRI scans, potentially reducing reporting time and streamlining the diagnostic process.

## Supplementary information

ELECTRONIC SUPPLEMENTARY MATERIAL

## Abbreviations

AP: Antero-posterior
APD: Antero-posterior diameter
AUC: Area under the receiver operating characteristic curve
CLAIM: Checklist for artificial intelligence in medical imaging
CSF: Cerebrospinal fluid
κw: Cohen’s quadratic weighted kappa score
CSA: Cross-sectional area
FSL: Fluid signal loss
IVD: Intervertebral disc
LCCS: Lumbar central canal stenosis
MRI: Magnetic resonance imaging
NPV: Negative predictive value
PPV: Positive predictive value
PCA: Principal component analysis
SD: Standard deviation
