# Supplementary material for: AI-based lumbar central canal stenosis classification on sagittal MR images is comparable to experienced radiologists using axial images
Source: Eur Radiol. 2024 Sep 20;35(4):2298–306. doi: 10.1007/s00330-024-11080-0 (PMC11913898; doi:10.1007/s00330-024-11080-0)
Supplement: Supplementary file 1 — ELECTRONIC SUPPLEMENTARY MATERIAL [file 330_2024_11080_MOESM1_ESM.pdf]

AI-based lumbar central canal stenosis classification on sagittal MR images is comparable to experienced radiologists using axial images

ELECTRONIC SUPPLEMENTARY MATERIAL

CLAIM 2024: Checklist for Artificial Intelligence in Medical Imaging

Section/Topic	No.	Item	Page No.
TITLE/ABSTRACT			
	1	Identification as a study of AI methodology, specifying the category of technology used (eg, deep learning)	1
ABSTRACT			
	2	Summary of study design, methods, results, and conclusions	1
INTRODUCTION			
	3	Scientific and/or clinical background, including the intended use and role of the AI approach	2
	4	Study aims, objectives, and hypotheses	2
METHODS			
Study Design	5	Prospective or retrospective study	3
	6	Study goal	2
Data	7	Data sources	3
	8	Inclusion and exclusion criteria	3
	9	Data preprocessing	4
	10	Selection of data subsets	3
	11	De-identification methods	3
	12	How missing data were handled	n/a
	13	Image acquisition protocol	3
Reference Standard	14	Definition of method(s) used to obtain reference standard	3
	15	Rationale for choosing the reference standard	3
	16	Source of reference standard annotations	3
	17	Annotation of test set	3
	18	Measures of inter- and intrarater variability of features described by the annotators	5
Data Partitions	19	How data were assigned to partitions	5
	20	Level at which partitions are disjoint	5
Testing Data	21	Intended sample size	5
Model	22	Detailed description of model	5
	23	Software libraries, frameworks, and packages	5
	24	Initialization of model parameters	5
Training	25	Details of training approach	4-5
	26	Method of selecting the final model	5
	27	Ensembling techniques	n/a
Evaluation	28	Metrics of model performance	5
	29	Statistical measures of significance and uncertainty	5
	30	Robustness or sensitivity analysis	n/a

	31	Methods for explainability or interpretability	n/a
	32	Evaluation on internal data	5
	33	Testing on external data	n/a
	34	Clinical trial registration	n/a
RESULTS			
Data	35	Numbers of patients or examinations included and excluded	3
	36	Demographic and clinical characteristics of cases in each partition	5
Model Performance	37	Performance metrics and measures of statistical uncertainty	6
	38	Estimates of diagnostic performance and their precision	6
	39	Failure analysis of incorrectly classified cases	6
DISCUSSION			
	40	Study limitations	7-8
	41	Implications for practice, including intended use and/or clinical role	7-8
OTHER INFORMATION			
	42	Provide a reference to the full study protocol or to additional technical details	n/a
	43	Statement about the availability of software, trained model, and/or data	n/a
	44	Sources of funding and other support; role of funders	1

Appendix B

Examples of midsagittal MRI slices from patients with epidural lesions. Images a-d show examples with accurate segmentation masks, whereas images e-h depict examples with mistakes in the segmentation masks. The images are zoomed in on the mistakes, highlighted with a white arrow.

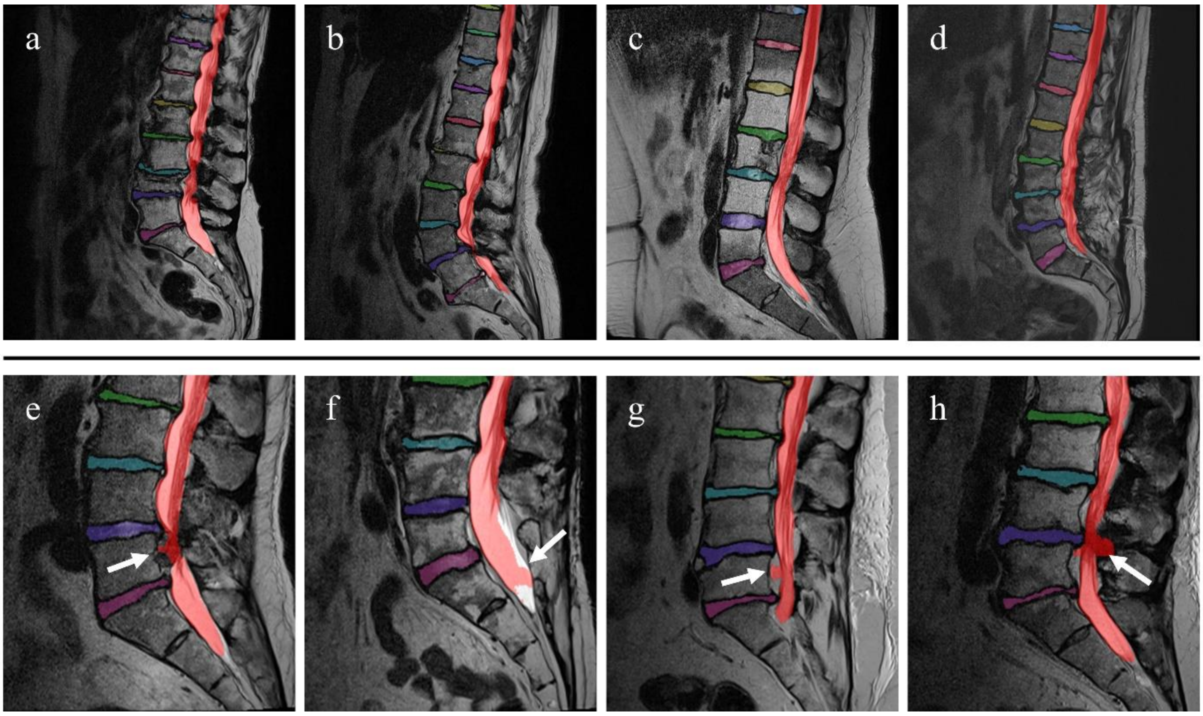

Appendix C

During the development phase, experiments were conducted with several machine learning models, including logistic regression, decision tree, and random forest classifiers. The decision tree and random forest classifiers' parameters were optimized using random grid search for both multigrade and binary classification. The performance of the classifiers for multigrade and binary classification models were evaluated using Cohen's weighted kappa and AUC scores respectively through 10-fold cross-validation.

The random forest and decision tree models were trained under specific settings, chosen after performing a random grid search. For the multiclass classification, the parameters were set to 1 and 9 samples per leaf and a maximum depth of 46 and 25 respectively for the random forest and decision tree models, and 208 trees for the random forest model. For binary classification the parameters were set to 23 and 20 sample per leaf and a maximum depth of 72 and 10 respectively for random forest and decision tree models, and 29 trees for the random forest model.

To compare the random forest, decision tree and logistic regression classifiers, the models were tested in 10-fold cross-validation and their differences were tested using a paired t-test. For both multigrade and binary classification, the random forest performed best, with a κw of 0.856 (95% CI: 0.830, 0.882) and an AUC of 0.978 (95% CI: 0.967, 0.989) respectively, while the decision tree and logistic regression models got a κw of 0.822 (95% CI: 0.793, 0.850) and 0.820 (95% CI: 0.791, 0.848) for multigrade classification and an AUC of 0.963 (95% CI: 0.949, 0.977) and 0.896 (95% CI: 0.958, 0.983) for binary classification respectively. The random forest model showed significantly superior performance to the decision tree and logistic regression models for multigrade classification, and to decision tree for binary classification. While reaching a higher mean AUC for binary classification, the random forest model did not show significant advantage over the logistic regression model. Since the random forest models showed the best performance, these were chosen to be compared to R3 and R4. All results are shown in the table below.

Multigrade models	κw (95% CI)	Difference	p-value
Random Forest	0.86 (0.83, 0.88)	-	-
Decision tree	0.82 (0.79, 0.85)	-0.04	0.012
Logistic regression	0.82 (0.79, 0.85)	-0.04	0.007
Binary models	AUC (95% CI)		
Random Forest	0.98 (0.97, 0.99)	-	-
Decision tree	0.96 (0.95, 0.97)	-0.015	0.008
Logistic regression	0.97 (0.96, 0.98)	-0.007	0.055
